# Artificial intelligence in oncology: current applications and future perspectives

**DOI:** 10.1038/s41416-021-01633-1

**Published:** 2021-11-26

**Authors:** Claudio Luchini, Antonio Pea, Aldo Scarpa

**Affiliations:** 1 Department of Diagnostics and Public Health, Section of Pathology, University and Hospital Trust of Verona, 37134 Verona, Italy; 2 ARC-Net Research Center, University and Hospital Trust of Verona, 37134 Verona, Italy; 3 Department of Surgery, the Pancreas Institute, University and Hospital Trust of Verona, 37134 Verona, Italy

**Keywords:** Cancer, Developing world

## Abstract

Artificial intelligence (AI) is concretely reshaping the landscape and horizons of oncology, opening new important opportunities for improving the management of cancer patients. Analysing the AI-based devices that have already obtained the official approval by the Federal Drug Administration (FDA), here we show that cancer diagnostics is the oncology-related area in which AI is already entered with the largest impact into clinical practice. Furthermore, breast, lung and prostate cancers represent the specific cancer types that now are experiencing more advantages from AI-based devices. The future perspectives of AI in oncology are discussed: the creation of multidisciplinary platforms, the comprehension of the importance of all neoplasms, including rare tumours and the continuous support for guaranteeing its growth represent in this time the most important challenges for finalising the ‘AI-revolution’ in oncology.

## Introduction

Artificial intelligence (AI) is concretely reshaping our lives and it is time to understand its evolution and achievements to model future development strategies. This is true also for oncology and related fields, where AI is now opening new important opportunities for improving the management of cancer patients, as will be highlighted in this perspective paper.

In 1950, Alan Turing was the first that conceives the idea of using computers to mimic intelligent behaviour and critical thinking [1]. In 1956, John McCarthy coined the term ‘artificial intelligence’ as ‘the science and engineering of making intelligent machines’ [1, 2]. AI began as a simple series of ‘if, then rules’, and has advanced in subsequent years for encompassing multifaceted and composite algorithms that perform similarly to the human brain [1].

Nowadays, AI represents an emerging and rapidly evolving model that regards different scientific fields, also those devoted to the management of cancer patients [2–5]. It can be seen as a general concept indicating the ability of a machine to learn and recognise patterns and interactions from a sufficient number of representative models, and to use this information for improving the current approach towards the process of decision-making in a specific field [3–5].

In precision oncology, AI is reshaping the existing scenario, aiming at integrating the large amount of data derived from multi-omics analyses with current advances in high-performance computing and groundbreaking deep-learning strategies [3]. Notably, the applications of AI are expanding and include new approaches for cancer detection, screening, diagnosis and classification, the characterisation of cancer genomics, the analysis of tumour microenvironment, the assessment of biomarkers with prognostic and predictive purposes and of strategies for follow-up and drug discovery [3–6].

For better understanding current roles and future perspectives of AI, two important terms/definitions, which are strictly associated with AI, should be enlightened: machine learning and deep learning. Machine learning is a general concept indicating the ability of a machine in learning and thus improving patterns and models of analysis, whereas deep learning indicates a machine-learning method that utilises complex and deep networks to finalise a highly predictive performance [3, 4]. Of note, these two concepts are central also in the AI revolution in the management of cancer patients.

Through a systematic review-based approach, we aim to clarify which are the current applications of AI in oncology-related fields, with a specific focus on already-approved devices. This approach will allow to better understand roles and potentialities of AI in the management of cancer patients, representing also a reliable point of start for discussing the most important future perspectives of AI in this field.

## Methods

**Fig. 1:**
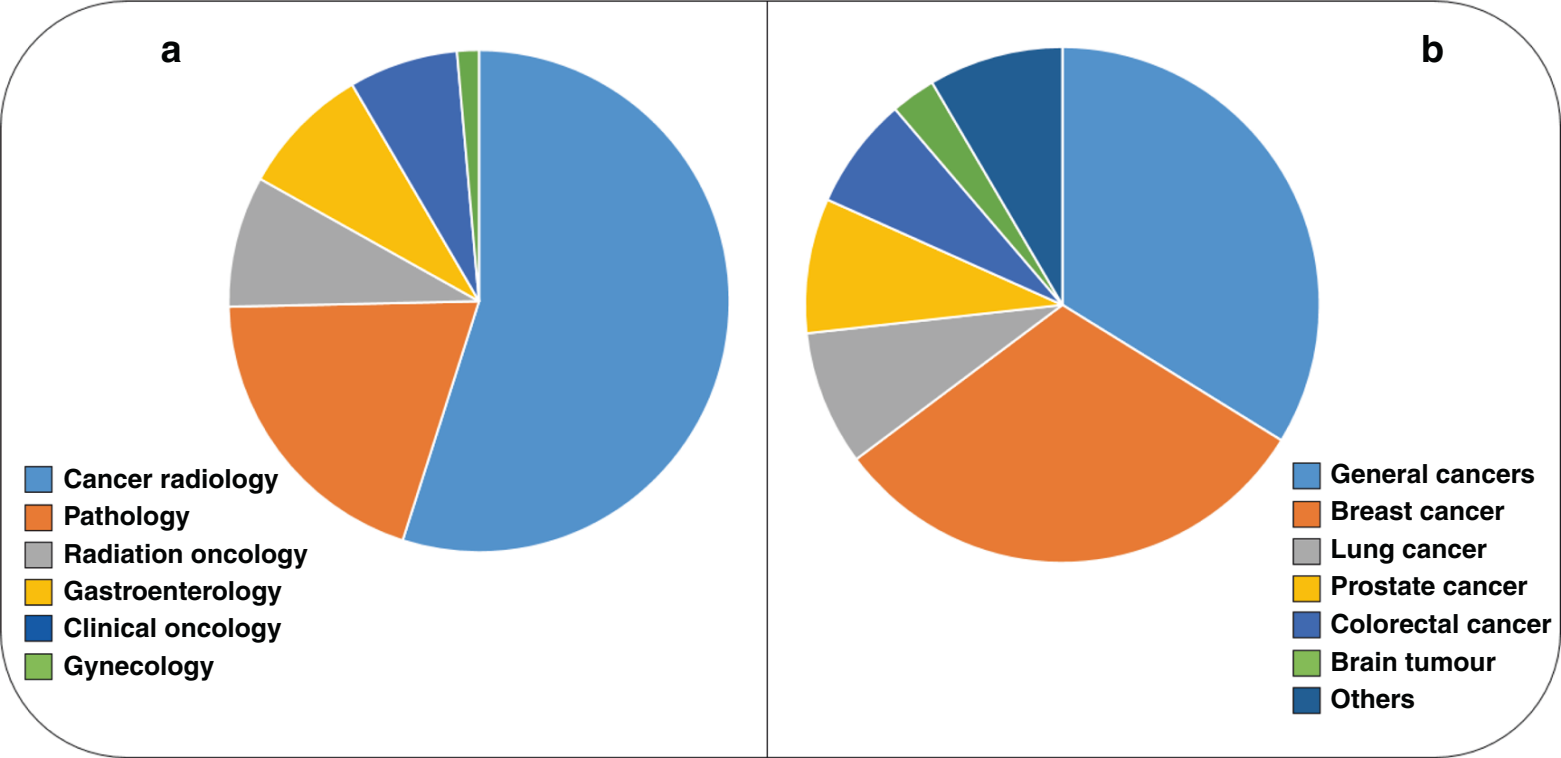
Current status of Artificial intelligence in oncology and related fields. Summarising representations of the artificial intelligence-based devices, FDA-approved, expressed by oncology-related specialties (**a**: cancer radiology 54.9%, pathology 19.7%, radiation oncology 8.5%, gastroenterology 8.5%, clinical oncology 7.0% and gynaecology 1.4%) and by tumour types (**b**: general cancers 33.8%, breast cancer 31.0%, lung cancer 8.5%, prostate cancer 8.5%, colorectal cancer 7.0% and brain tumours 2.8%, others: 6 tumour types, 1.4% each).

The systematic review-based approach adhered to the PRISMA statement preset protocol [7]. For providing a comprehensive portrait of the current situation of the roles played by AI in the management of cancer patients, a systematic review was performed, investigating the AI-based devices that have already obtained an official approval for entering into clinical practice in oncology and its related fields. To this aim, two authors (C.L. and A.P.) retrieved all AI-based devices that have obtained the Federal Drug Administration (FDA) approval in oncology-related fields, extracting all potential data by searching FDA official databases (https://www.fda.gov/downloads/medicaldevices/deviceregulationandguidance/guidancedocuments/ucm514737.pdf; https://www.fda.gov/media/145022/download; https://www.accessdata.fda.gov/scripts/cdrh/cfdocs/cfPMN/denovo.cfm; https://www.accessdata.fda.gov/scripts/cdrh/cfdocs/cfPMA/pma.cfm; https://www.accessdata.fda.gov/scripts/cdrh/cfdocs/cfpmn/pmn.cfm. Last access for all documents: 05/31/2021. Such data were also integrated with all previous related reviews or commentaries. All data were organised to be separately presented by the specific oncologic areas in the text, as well as in a summary figure (Fig. 1).

## Results

Altogether, the search documented the presence of 71 AI-associated or AI-associable devices that have already received an official FDA approval (Table 1), matching data also from previous related reviews [2, 8–10]. The oncology-related field that counts for the largest number of AI devices is cancer radiology, with the majority of approved devices (54.9%). It is followed by pathology (19.7%), radiation oncology (8.5%), gastroenterology (8.5%), clinical oncology (7.0%) and gynaecology 1 (1.4%) (Table 1, Fig. 1a). The vast majority of the approved devices (>80%) regarded the complex area of cancer diagnostics.

**Table 1:** List of AI-associated/associable equipped medical devices approved by the US FDA specifically for oncology-related fields.

N°	Month of approval	Name of the device	Description of the device and its role	Specific area of interest
1	February 2015	ER APP, Breast Cancer (Visiopharm A/S)	Determination of oestrogen receptor positivity and negativity in breast cancer	Pathology
2	February 2015	PR APP, Breast Cancer (Visiopharm A/S)	Determination of progesterone receptor positivity and negativity in breast cancer	Pathology
3	August 2015	Kaiku Health (Kaiku Oy)	Outcome monitoring and symptom tracking for cancer patients	Clinical Oncology
4	November 2015	ClearRead CT (Riverain Technologies LLC.)	Assistance in review of multi-slice computed tomography exams of the chest and detection of potential nodules that radiologist should review	Cancer Radiology
5	December 2015	Transpara (ScreenPoint Medical BV)	Reading aid for physicians interpreting screening mammograms to identify regions suspicious for breast cancer	Cancer Radiology
6	June 2016	SmartTarget (SmartTarget Ltd.)	Image-guided interventional and diagnostic procedures involving the prostate gland	Cancer Radiology
7	September 2016	Eclipse Treatment Planning System V15.6 (arian Medical Systems Inc.)	Radiotherapy treatment planning for patients with malignant or benign diseases	Radiation Oncology
8	August 2016	LungQ (Thirona Corp.)	Support in diagnosis and documentation of pulmonary tissues images (eg, abnormalities) from CT thoracic datasets	Cancer Radiology
9	March 2017	ColonFlag (Medial EarlySign Inc.)	High risk colorectal cancer detection support for pre-symptomatic patients	Gastroenterology
10	May 2017	AmCAD-US (AmCad BioMed Corporation)	A software to visualise and quantify ultrasound image data with backscattered signals.	Cancer Radiology
11	June 2017	C the Signs (C the Signs Ltd.)	Assessment of symptoms to support cancer diagnosis	Clinical Oncology
12	July 2017	QuantX (Quantitative Insights)	An AI-equipped diagnosis system to aid in accurate diagnosis of breast cancer.	Cancer Radiology
13	December 2017	Veye Chest (Aidence BV)	Pulmonary nodule detection support from CT scans	Cancer Radiology
14	January 2018	Arterys Oncology DL (Arterys)	An AI-based, cloud-based medical imaging software that automatically measures and tracks lesions and nodules in MRI and CT.	Cancer Radiology
15	January 2018	GI Genius (Medtronic Inc. (parent company: Medtronic plc.))	Colorectal cancer detection support	Gastroenterology
16	January 2018	QVCAD (QView Medical Inc.)	Aid to detect mammography-occult lesions in regions not known to have suspicious findings	Cancer Radiology
17	February 2018	DLCExpert (Mirada Medical Ltd.)	Contouring assistance for radiation therapy from CT scans	Radiation Oncology
18	May 2018	HealthMammo (Zebra Medical Vision Inc.)	Mammograms processed and analysed for suspected breast cancer lesions	Cancer Radiology
19	July 2018	Arterys Oncology DL (Arterys Inc.)	Support of oncological workflow by helping user confirm absence or presence of lesions; application supports anatomical datasets, such as CT or MR	Cancer Radiology
20	October 2018	Hot Spot APP (Visiopharm A/S)	Hotspot scoring method for various cancer applications	Pathology
21	October 2018	Invasive Tumour Detection APP (Visiopharm A/S)	Cytokeratin and p63 marker assessment for invasive and non-invasive tumour distinguishment	Pathology
22	October 2018	AmCAD-UT (AmCad BioMed Corporation)	Assistance in analysis of thyroid ultrasound images	Cancer Radiology
23	October 2018	Mia -Mammography Intelligent Assessment (KheironMedical Technologies Ltd.)	Breast cancer detection support from mammograms	Cancer Radiology
24	October 2018	Arterys MICA (Arterys)	An AI-based platform for analysing medical images such as MRI and CT.	Cancer Radiology
25	November 2018	SubtlePET (Subtle Medical)	An AI-powered technology that enables centers to deliver a faster and safer patient scanning experience, while enhancing exam throughput and provider profitability.	Cancer Radiology
26	February 2019	DERM (Skin Analytics Ltd.)	Skin cancer diagnosis support	Clinical Oncology
27	February 2019	ART-Plan.annotate (heraPanacea SAS)	Contouring of tumour and surrounding organs for radiotherapy	Radiation Oncology
28	March 2019	cmTriage (CureMetrix)	An AI-based triage software for mammography.	Cancer Radiology
29	April 2019	Deep Learning Image Reconstruction (GE Medical Systems)	A deep-learning-based CT image reconstruction technology.	Cancer Radiology
30	April 2019	Auto Lung Nodule Detection (Samsung Electronics Co. Ltd. (parent company: Samsung Group)	Lung nodule detection for diagnostic support from X-ray images	Cancer Radiology
31	May 2019	JPC-01K (JLK Inspection Inc.)	Prostate cancer detection for diagnostic support from MRI images	Cancer Radiology
32	May 2019	syngo.Breast Care (Siemens Healthcare GmbH (parent company: Siemens AG))	Reading and reporting for diagnostic support from mammograms	Cancer Radiology
33	June 2019	Aquilion ONE (TSX-305A/6) V8.9 with AiCE (Canon MedicalSystems Corporation)	A device to acquire and display cross-sectional volumes of the whole body, including the head, with the capability of imaging whole organs in a single rotation.	Cancer Radiology
34	July 2019	ProFound AI for 2D Mammography (iCAD Inc.)	Breast cancer detection assistance and workflow solution from 2D mammograms	Cancer Radiology
35	July 2019	ProFound AI for Digital Breast Tomosynthesis (iCAD Inc.)	Computer-assisted detection and diagnosis (CAD) software device intended to be used while reading digital breast tomosynthesis (DBT) exams	Cancer Radiology
36	July 2019	RayCare 2.3 (RaySearch Laboratories)	An oncology information system used to support workflows, scheduling and clinical information management for oncology care and follow-up.	Cancer Radiology
37	August 2019	Ethos Radiotherapy Treatment (Varian Medical Systems Inc.)	Managing and monitoring radiation therapy treatment plans and sessions	Radiation Oncology
38	September 2019	AVEC (Automated Visual Evaluation of the Cervix) (MobileODT Ltd.)	Cervical cancer screening support for diagnostic support	Gynecology
39	September 2019	Breast-SlimView (Hera-MI SAS)	Breast cancer detection for diagnostic support from mammograms	Cancer Radiology
40	September 2019	Vara (Merantix Healthcare GmbH)	Breast cancer screening support and triaging from mammograms	Cancer Radiology
41	October 2019	ProFound AI Software V2.1 (iCAD)	A CAD software device intended to be used concurrently by interpreting physicians while reading DBT	Cancer Radiology
42	October 2019	DeepDx-Prostate Connect (Deep Bio Inc.)	Recognition of acinar adenocarcinoma of the prostate	Pathology
43	November 2019	Paige Prostate (Paige Inc.)	Cancer detection in prostate needle biopsies	Pathology
44	November 2019	Paige Insight (Paige Inc.)	Digital pathology viewer for diagnostic support	Pathology
45	December 2019	Transpara (ScreenPoint Medical)	A device for use as a concurrent reading aid for physicians interpreting screening mammograms from compatible FFDM systems to identify regions suspicious for breast cancer and assess their likelihood of malignancy.	Cancer Radiology
46	December 2019	QyScore software (Qynapse SAS)	Automatic labelling, visualisation and volumetric quantification of segmentable brain structures and lesions from MR images	Cancer Radiology
47	December 2019	Discovery AI (Pentax Medical GmbH (parent company: Pentax Corporation))	Polyp detection support during a colorectal examination	Gastroenterology
48	December 2019	RayStation (RaySearch Laboratories AB)	Treatment planning and analysis of radiation therapy	Radiation Oncology
49	December 2019	RayCare 2.3 (RaySearch Laboratories AB)	Support of workflows, scheduling and clinical information management for oncology care and follow-up	Clinical Oncology
50	January 2020	JBD-01K (JLK Inspection Inc.)	Breast cancer detection for diagnostic support from mammograms	Cancer Radiology
51	January 2020	AI-Pathway Companion Prostate Cancer (Siemens Healthcare GmbH (parent company: Siemens AG)	Prostate cancer detection for diagnostic support	Clinical Oncology
52	January 2020	MRCAT Brain (Philips Medical Systems MR Finland (parent company: Philips NV))	Radiation therapy planning through automated image segmentation for brain tumour patients	Radiation Oncology
53	February 2020	InferRead CT Lung (Beijing Infervision Technology Co. Ltd.)	Lung cancer screening and management tool from CT scans	Cancer Radiology
54	February 2020	b-box (b-rayZ GmbH)	Assessment of mammography image quality and breast density from mammograms	Cancer Radiology
55	February 2020	Metastasis Detection App (Visiopharm A/S)	Metastasis detection in lymph nodes for colorectal and breast adenocarcinoma	Pathology
56	February 2020	Galen Prostate (Ibex Medical Analytics Ltd)	Identification of suspected cancer on prostate core needle biopsies	Pathology
57	February 2020	densitasAI (Densitas Inc.)	Breast density assessment support from mammograms	Cancer Radiology
58	March 2020	Broncholab (Fluidda Inc)	Support in diagnosis and documentation of pulmonary tissue images(eg, abnormalities) from CT thoracic datasets	Cancer Radiology
59	March 2020	Syngo.CT Lung CAD (Siemens Medical Solutions Inc. (parent company: Siemens AG))	Assistance in detection of solid pulmonary nodules during review of multi-detector computed tomography examinations of the chest	Cancer Radiology
60	March 2020	MammoScreen (Therapixel SA)	Help to identify findings on screening FFDM acquired with compatible mammography systems and assess level of suspicion	Cancer Radiology
61	March 2020	CAD EYE (FUJIFILM Europe GmbH)	Colonic polyps detection and characterisation support during a colonoscopy	Gastroenterology
62	May 2020	NaviCam Capsule Endoscope System with NaviCam Stomach Capsule (AnX Robotica, Inc.)	A magnetically maneuvered capsule endoscopy system consists of an ingestible capsule and magnetic controller and is used for visualisation of the stomach and duodenum. The magnetic controller is used outside of the patient and is magnetically coupled with the capsule to control its location and viewing direction.	Gastroenterology
63	June 2020	Cobas® EZH2 Mutation Test (Roche Molecular System, Inc.)	The test is intended for the identification of follicular lymphoma patients with an EZH2 mutation for treatment with TAZVERIK (tazemetostat); coupled with the cobas z 480 analyzer.	Pathology
64	July 2020	Her2 dual ish dna probe cocktail	It is intended to determineHER2 gene amplification status by enumeration of the ratio of the HER2 gene to Chromosome 17 by light microscopy.	Pathology
65	October 2020	Cintec plus cytology (Ventana Medical Systems, Inc.)	Qualitative immunocytochemical assay for the simultaneous detection of the p16INK4a and Ki-67 proteins in cervical specimens, intended for the diagnosis of cervical cancer.	Pathology
66	November 2020	Genius AI Detection (Hologic, Inc.)	Software device intended to identify potential abnormalities in breast tomosynthesis images	Cancer Radiology
67	November 2020	FoundationOne Liquid CDx (Foundation Medicine, Inc.)	It is a qualitative NGS-based test interrogating 311 genes. It utilises circulating cell-free DNA (cfDNA) isolated from plasma of cancer patients, and is intended to be used as a companion diagnostic to identify patients who may benefit from treatment with targeted therapies (targets identified with NGS)	Pathology
68	January 2021	Visage Breast Density (Visage Imaging)	The software application is intended for use with compatible full-field digital mammography to aid radiologists in the assessment of breast tissue composition	Cancer Radiology
69	January 2021	Imagio Breast Imaging System (Seno Medical Instruments, Inc.)	Allows an improved classification of breast masses compared to ultrasound alone; includes an AI-based software.	Cancer Radiology
70	April 2021	VENTANA MMR RxDx Panel (Ventana Medical Systems, Inc.)	CDx for identifying patients with endometrial cancer with dMMR status who may benefit from treatment with Jemperli (dostarlimab-gxly).	Pathology
71	April 2021	GI Genius (Cosmo Artificial Intelligence—AI, LTD)	It is a computer-assisted reading tool designed to aid endoscopists in detecting colonic mucosal lesions (such as polyps and adenomas) in real time during standard white-light endoscopy.	Gastroenterology

Summary of the different oncology-related medical areas of all AI-associated devices approved by FDA: 39 cancer radiology (54.9%); 14 pathology (19.7%); 6 radiation oncology (8.5%); 6 gastroenterology (8.5%); 5 clinical oncology (7.0%), gynecology 1 (1.4%).

Summary of the different tumour types investigated by the presented devices: 24 general cancers (33.8%); 22 breast cancer (31.0%); 6 lung cancer (8.5%); 6 prostate cancer (8.5%); 5 colorectal cancer (7.0%); 2 brain tumours (2.8%); 6 others (6 types): 1.4% each.

*AI* artificial intelligence, *US FDA* United States Food and Drug Administration, *CT* computed tomography, *MRI* magnetic resonance imaging, *ECG* electrocardiogram, *CAD* computer-aided detection/diagnosis, *DBT* digital breast tomosynthesis, *FFDM* full-field digital mammography.

Regarding the different tumour types that can be investigated by adopting such devices, the majority of them has been conceived for being applied to a wide spectrum of solid malignancies (cancer in general, 33.8%). The specific tumour that counts for the largest number of AI devices is breast cancer (31.0%), followed by lung and prostate cancer (8.5% each), colorectal cancer (7.0%), brain tumours (2.8%) and others (6 types, 1.4% each) (Table 1, Fig. 1b).

## Discussion and future perspectives

In this paper, a comprehensive overview on current applications of AI in oncology-related areas is provided, specifically describing the AI-based devices that have already obtained the official approval to enter into clinical practice. Starting from its birth, AI demonstrated its cross-cutting importance in all scientific branches, showing an impressive growth potential for the future. As highlighted in this study, this growth has interested also oncology and related specialties.

In general, the application of the FDA-approved devices has not been conceived as a substitute of classical analysis/diagnostic workflow, but is intended as an integrative tool, to be used in selected cases, potentially representing the decisive step for improving the management of cancer patients. Currently, in this field, the branches where AI is gaining a larger impact are represented by the diagnostic areas, which count for the vast majority of the approved devices (>80%), and in particular radiology and pathology.

Cancer diagnostics classically represents the necessary point of start for designing appropriate therapeutic approaches and clinical management, and its AI-based refining represents a very important achievement. Furthermore, this indicates that future developments of AI should also consider unexplored but pivotal horizons in this landscape, including drug discovery, therapy administration and follow-up strategies. In our opinion, for determining a decisive improvement in the management of cancer patients, indeed, the growth of AI should follow comprehensive and multidisciplinary patterns. This represents one of the most important opportunities provided by AI, which will allow the correct interactions and integration of oncology-related areas on a specific patient, rendering possible the challenging purposes of personalised medicine.

The specific cancer types that now are experiencing more advantages from AI-based devices in clinical practice are first of all breast cancer, lung cancer and prostate cancer. This should be seen as the direct reflection of their higher incidence compared with other tumour types, but in the future, additional tumour types should be taken into account, including rare tumours that still suffer from the lack of standardised approaches. Since AI is based on the collection and analysis of large datasets of cases, however, the improvement in the treatment of rare neoplasms will likely represent a late achievement. Notably, if together considered, rare tumours are one of the most important category in precision oncology [11]. Thus, in our opinion, ongoing strategies of AI development cannot ignore this tumour group; although the potential benefits seem far away, it is already time to start collecting data on rare neoplasms.

One of the most promising expectancy for AI is the possibility to integrate different and composite data derived from multi-omics approaches to oncologic patients. The promising tools of AI could be the only able to manage the big amount of data from different types of analysis, including information derived from DNA and RNA sequencing. Along this line, the recent release of American College of Medical Genetics standards and guidelines for the interpretation of the sequence variants [12] has fostered a new wave of AI development, with innovative opportunities in precision oncology (https://www.businesswire.com/news/home/20190401005976/en/Fabric-Genomics-Announces-AI-based-ACMG-Classification-Solution-for-Genetic-Testing-with-Hereditary-Panels; last access 09/21/2021). In our opinion, however, the lack of ground-truth information derived from protected health- data repositories still represents a bottleneck in evaluating the accuracy of AI applications for clinical decision-making.

Overall considered, AI is providing a growing impact to all scientific branches, including oncology and its related fields, as highlighted in this study. For designing new development strategies with concrete impacts, the first steps are representing by knowing its historical background and understanding its current achievements. As here highlighted, AI is already entered into the oncologic clinical practice, but continuous and increasing efforts should be warranted to allow AI expressing its entire potential. In our opinion, the creation of multidisciplinary/integrative developmental views, the immediate comprehension of the importance of all neoplasms, including rare tumours and the continuous support for guaranteeing its growth represent in this time the most important challenges for finalising the ‘AI-revolution’ in oncology.

## Data Availability

All data are available in the paper.
